# Mealtime behavior among siblings and body mass index of 4–8 year olds: a videotaped observational study

**DOI:** 10.1186/s12966-015-0256-7

**Published:** 2015-07-15

**Authors:** Rana H. Mosli, Alison L. Miller, Niko Kaciroti, Karen E. Peterson, Katherine Rosenblum, Ana Baylin, Julie C. Lumeng

**Affiliations:** Department of Nutritional Sciences, School of Public Health, University of Michigan, Ann Arbor, MI USA; Clinical Nutrition Department, Faculty of Applied Medical Sciences, King Abdulaziz University, Jedddah, Kingdom of Saudi Arabia; Department of Health Behavior and Health Education, School of Public Health, University of Michigan, Ann Arbor, MI USA; Center for Human Growth and Development, University of Michigan, Ann Arbor, MI USA; Department of Nutrition, Harvard School of Public Health, Boston, MA USA; Department of Psychiatry, University of Michigan, Ann Arbor, MI USA; Department of Epidemiology, School of Public Health, University of Michigan, Ann Arbor, MI USA; Department of Pediatrics and Communicable Diseases, University of Michigan, Ann Arbor, MI USA

**Keywords:** Birth order, Siblings, Body Mass Index, Family, Videotape Recording, Meals

## Abstract

**Background:**

Being a last-born child and having a sister have been associated with higher body mass index (BMI). Encouragement to eat that overrides children’s self-regulation has been reported to increase the risk of obesogenic eating behaviors. This study sought to test the hypothesis that encouragement to eat during mealtime from older siblings and sisters mediates associations of being last-born or having a sister with higher BMI.

**Methods:**

Children aged 4–8 years (*n* = 75) were videotaped while eating a routine evening meal at home with one sibling present. Encouragement to eat (defined as direct prompts to eat or general positive statements about food) delivered to the index child (IC) from the sibling was coded from the videotape. Path analysis was used to examine associations between IC’s birth order, sibling’s sex, encouragement counts, and IC’s measured BMI z-score (BMIz).

**Results:**

Being the younger sibling in the sibling dyad was associated with the IC receiving more encouragements to eat from the sibling (β: 0.93, 95 % confidence interval (CI): 0.59, 1.26, *p* < 0.0001). The IC having a sister compared with a brother was not associated with the IC receiving more encouragements to eat from the sibling (β: 0.18, 95 % CI: −0.09, 0.47, *p* = 0.20). The IC receiving more encouragements to eat from the sibling was associated with lower IC BMIz (β: −0.06, 95 % CI: −0.12, 0.00, *p* = 0.05).

**Conclusions:**

Children were more likely to receive encouragements to eat from older siblings than younger siblings. Being the recipient of encouragements to eat from a sibling was associated with lower, not higher, child BMIz, which may reflect sibling modeling of maternal behavior. Future longitudinal studies are needed to examine whether encouragements to eat from siblings lead to increase in BMI over time. Encouragements from siblings may be a novel intervention target for obesity prevention.

## Background

Childhood obesity continues to be a public health concern [1]. Family-based interventions have shown promise for childhood obesity prevention, though as with other obesity intervention strategies, effects tend to be modest [2]. Careful examination of interaction patterns between family members that may contribute to childhood obesity risk could provide novel targets for refining and strengthening the effectiveness of family-based interventions. The family mealtime is often used as a venue for studying family interaction patterns and has also been a key focus of childhood obesity prevention programs [3–5]. Most studies examining features of family mealtimes and childhood obesity have focused on mother-child interactions or the mealtime environment [5–11]. There is a lack of understanding of how siblings interact during mealtimes and how different interaction patterns relate to child body mass index (BMI).

**Fig. 1:**
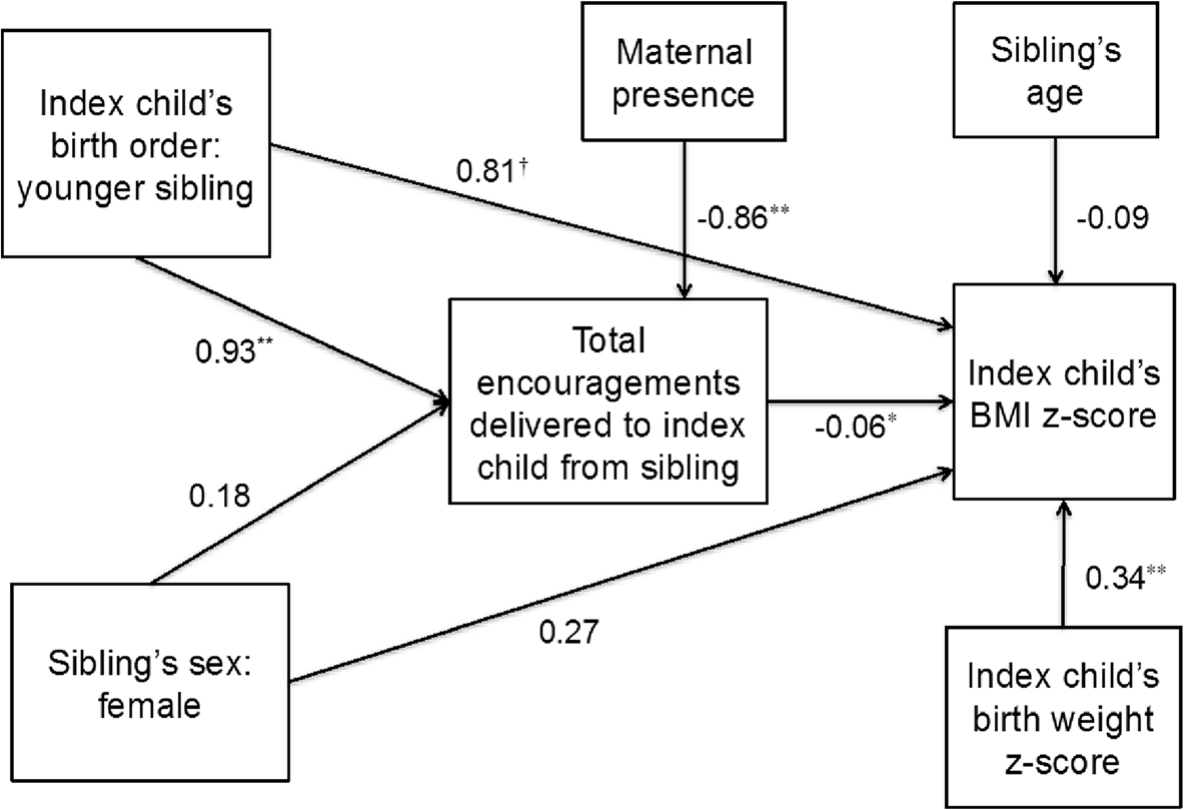
Path model showing path coefficients for associations between index child’s birth order, sibling’s sex, total encouragements delivered to index child from sibling, and index child’s BMI z-score. * *p* ≤ 0.05 ** *p* ≤ 0.01 **†** *p* ≤ 0.1

Interactions between siblings during childhood can influence development and behavior [12] through caregiving and role modeling interactions [12–14]. During mealtimes, sibling caregiving or role modeling behaviors may be observed as encouragements to eat. Although some studies found that mothers encourage their children to eat as a response to lower child BMI [15], others suggest that maternal encouragement to eat is a predictor of child weight status, such that encouragements to eat may override the child’s ability to respond to internal satiety cues and lead to increased risk of obesity or obesity-promoting eating behaviors [10, 16, 17]. Mothers encouraging children to eat has been a frequent target for obesity prevention efforts. However, we have been unable to identify any published studies examining the potential role of siblings encouraging a child to eat in shaping children’s eating behavior and obesity risk.

The child’s birth order and sex of siblings shape the nature of interactions between the siblings [12]. Older siblings and sisters more often act as caregivers and role models for their siblings than do younger siblings and brothers and thus may be more likely to encourage their siblings to eat [12, 14, 18]. We and others have previously reported that children who are the youngest in a sibship are more likely to be obese [19–22] and that having a sister, compared with a brother, is associated with greater likelihood of being overweight [23, 24]. Prior work has not yet identified a mechanism for this association [19, 20, 24]. The objective of this study was therefore to test the hypothesis that encouragement to eat initiated by older siblings and sisters is an underlying process for the association of being a younger sibling and having a sister with higher BMI.

## Methods

### Participants and procedures

The study sample includes 301 child-mother dyads recruited through Head Start programs in South Central Michigan. Head Start is a federally funded preschool program for low-income, high-risk families in the United States (US). Participants were drawn from a longitudinal cohort initiated in 2009 to investigate associations between stress and eating among low-income children. Children described in this study were between the ages of 4 and 8 years at the time of data collection. Inclusion criteria were: caregiver is fluent in English and does not have a college degree; and child is not in foster care, has no serious medical problems or history of food allergies and was born at ≥ 35 weeks gestation without significant perinatal or neonatal complications. For this analysis we only included children who were living with their biological mothers (as this represents the majority of this sample), who were living with only one sibling, and who had complete data on all variables (*n* = 102). Of those 102 children, we only included index children whose siblings were at least a year old (*n* = 86) on the premise that the processes via which infants may influence eating behavior of siblings could be fundamentally different. Mothers provided written informed consent for themselves and for their children. The University of Michigan Institutional Review Board approved this study.

During two study visits, mothers completed questionnaires, and trained staff members obtained child anthropometry. Three videotaped home mealtime observations were completed for each family. Each mother was asked to record three routine evening meals within a single week. Research assistants called each mother after the meal to obtain information regarding individuals present. These family mealtime observations (FMOs) followed standard procedures that have been previously described [25].

For the present study, inclusion criteria for the FMO videotape included that the index child (IC) was eating with his/her sibling, and that the IC was not eating with other children in addition to the sibling. We systematically selected one of the three FMO videos for each IC. We started video selection with the second FMO video on the premise that we would expect families to be more acclimated to the camera by the second home observation. If the second FMO video did not meet the inclusion criteria, we then assessed the third FMO video; if the third FMO video did not meet inclusion criteria, we assessed the first FMO video. After assessment of the FMO videos for each IC, a final sample of 75 index children was identified (8 from the first FMO, 55 from the second FMO, and 12 from the third FMO). The sample included in this analysis (*n* = 75) did not differ from the sample not included (*n* = 226) with regard to child sex, child race/ethnicity, birth weight z-score, and maternal age.

### Measures

#### Demographic characteristics

Mothers reported information regarding IC’s birthdate, sex, and race/ethnicity (dichotomized for this report as non-Hispanic white vs. not) and mother’s birthdate and years of education (dichotomized as more than or equal to a high school education vs. not). Birthdates and dates of visits were used to calculate child and maternal age.

### Sibling characteristics and birth order

For each individual living in the household, as well as for each individual on the FMO videotapes, mothers reported age, sex, and relationship to the IC. This information was used to determine the IC’s birth order (i.e., younger sibling vs. older sibling) and characteristics of the siblings.

### Coding of interactions between index child and sibling

To evaluate mealtime sibling behaviors that may be most relevant to child obesity risk, we developed a coding scheme based on Bob and Tom’s Method of Assessing Nutrition (BATMAN) [10]. The BATMAN is an observational assessment used to evaluate parental behavior around food [10]. Although restrictive feeding behaviors are part of the BATMAN, we did not code these behaviors as they were not observed to occur between siblings with meaningful frequency. Although the BATMAN distinguishes between physical and verbal encouragements to eat, we did not observe frequent physical encouragements to eat between siblings and therefore focused our coding scheme on verbal encouragements to eat. The BATMAN defines verbal encouragements to eat as suggesting, demanding, directing, and making positive statements about food. We adapted some of the operational definitions to be consistent with theoretically important features of sibling interactions (i.e., parent-like interactions or “complementarity” and peer-like interactions or “reciprocity”) [12]. For example, food offers (representing complementarity) and statements about eating/finishing the food (representing reciprocity) were counted as verbal encouragements to eat.

Encouragements to eat delivered by the sibling and directed to the IC were coded in 5-min intervals from the videos. Ten percent of the videos were double coded and inter-rater reliability by intraclass correlation coefficient exceeded 0.80. Number of encouragements was summed across intervals to create the variable “total encouragements delivered to IC by sibling”.

### Mealtime maternal presence

Siblings interact differently when their mother is present [26, 27]. In order to adjust for maternal presence, we coded whether the mother was sitting with the siblings during the meal in each 5-min interval (yes vs. no for each interval). Inter-rater reliability computed as Cohen’s kappa was 1.00. We created the variable “proportion of intervals in which mother is present” by dividing the total number of intervals in which the mother was sitting with the siblings by the total number of intervals.

### Anthropometry

Staff members measured index children’s weight and height during study visits using standardized procedures. BMI was calculated and age and sex specific BMI z-score (BMIz) for the IC was calculated based on the US Centers for Disease Control and Prevention reference growth curves [28]. Mothers reported the IC’s birth weight, which was converted to z-scores based on National Centers for Health Statistics Natality Datasets [29]. Birth weight z-scores were missing and were imputed for 26 subjects using multiple imputation.

### Statistical analysis

We conducted statistical analysis using Stata version 13 (StataCorp. 2013. *Stata Statistical Software: Release 13*. College Station, TX: StataCorp LP). First, we calculated descriptive statistics for sample characteristics. Then, to test our hypothesis that encouragements to eat from the sibling is a mediating variable in the association of IC’s birth order and the sibling’s sex with IC’s BMIz, we conducted path analysis, which is an extension of the regression model comprised only of directly observed variables [30]. We ran our path model testing associations between IC’s birth order, the sibling’s sex, encouragements to eat directed to the IC from the sibling, and IC’s BMIz. We included the binary variables IC’s birth order (with “older sibling” as the reference category) and sibling’s sex (with “male” as the reference category) as predictors in the model. A Poisson distribution was used to model the mediating count variable “total encouragements delivered to IC from sibling”, and “number of meal intervals” was set as the offset variable to account for variations in length of the meal. The model was adjusted for maternal presence (i.e., proportion of intervals in which mother is present), sibling’s age, and the IC’s birth weight z-score. For all statistical analyses, significance level was set at 0.05.

## Results

Mean IC age was 5.3 years (± SD 0.8), and about half (50.70 %) were male (Table 1). Path analysis showed that the IC being the younger sibling in the dyad, as opposed to the older sibling, was associated with receiving more encouragements to eat from the sibling (β: 0.93, 95 % CI: 0.59, 1.26, *p* < 0.0001). The IC having a sister as opposed to a brother was not directly associated with the IC receiving more encouragements to eat from the sibling (β: 0.18, 95 % CI: −0.09, 0.47, *p* = 0.20). The IC receiving more encouragements to eat from the sibling was associated with lower IC BMIz (β: −0.06, 95 % CI: −0.12, 0.00, *p* = 0.05). There was a marginally significant direct positive association between the IC being the younger sibling in the sibling dyad and the IC’s BMIz (β: 0.81, 95 % CI: −0.82, 1.70, *p* = 0.08). There was no direct association of the IC having a sister, as opposed to a brother, with the IC’s BMIz (β: 0.27, 95 % CI: −0.17, 0.72, *p* = 0.23) (Fig. 1).

**Table 1:** Sample characteristics^a^

	Total
	*n* = 75
Index child age, M(SD)	5.33 (0.79)
Index child sex, n (%)	
Male	38 (50.70)
Female	37 (49.30)
Index child race/ethnicity, n (%)	
Non-Hispanic white	44 (58.70)
Hispanic or not white	31 (41.30)
Maternal age, M (SD)	30.85 (6.73)
Maternal education, n (%)	
≤ High school education	31 (41.3)
> High school education	44 (58.7)
Sibling age, M (SD)	6.14 (3.49)
Sibling sex, n (%)	
Male	37 (49.3)
Female	38 (50.7)
Index child birth order, n (%)	
Younger sibling	41 (54.7)
Older sibling	34 (45.3)
Total encouragements delivered to index child from sibling, M(SD)	2.81 (3.93)
Proportion of intervals in which mother is present, M(SD)	0.86 (0.30)
Index child BMI z-score, M(SD)	0.81 (1.08)
Index child birth weight z-score, M(SD)	−0.22 (1.03)

^**a**^ Table showing means (M) and standard deviations (SD) or counts (n) and percentages (%)

## Discussion

Findings from this study did not support our hypothesized conceptual model that receiving more encouragements to eat from a sibling is an underlying process for the association between having an older sibling or a sister with higher child BMIz. However, our results do provide support for our hypothesis that siblings play an important role in the family mealtime environment.

Our study suggests that birth order is associated with the number of encouragements a child receives from his/her sibling, with younger siblings receiving more encouragements to eat from their older siblings. We did not detect a statistically significant association between having a sister and receiving more encouragements to eat, though the direction of association was in the expected direction. In summary, in consensus with some of the available literature on sibling interactions in other domains, we found that older siblings may act as potent caregivers and role models during mealtimes [12, 14, 18]. These novel findings regarding how siblings interact around food may contribute to better understanding of how families function during mealtimes.

Contrary to our hypothesis that encouragements to eat directed to the IC from the sibling would be positively associated with the IC’s BMIz, we found that encouragements to eat directed to the IC from the sibling was associated with the IC having a lower BMIz. We had based our hypothesis on reports that encouragement to eat from mother to child was positively associated with child overweight [10, 16, 17]. However, others have reported that controlling maternal feeding practices (including encouragement to eat) are inversely associated with child BMI and are a reaction to the child’s weight status [15, 31–33]. It is thus not fully understood whether controlling feeding behaviors and encouragements to eat by parents are associated with lower concurrent BMI, or whether they may predict increases in BMI prospectively [6–8, 15, 31, 34]. Since mothers may encourage children who are perceived to be thinner or have a poorer appetite to eat more [15, 32], it is plausible that this kind of feeding behavior might over time reduce the child’s ability to self-regulate intake in response to satiety cues and eventually lead to excessive weight gain [6–8]. Whether or not this is the case with regard to the association between encouragements from siblings and child BMI is unknown. However, our data suggest that cross-sectionally, older siblings may be imitating their mothers and encouraging siblings who are thinner to eat more. Prospective studies are needed to better establish the direction of this association.

Strengths of this study include the use of an observational assessment of interactions between siblings during a naturalistic mealtime setting. Limitations of this study include the small sample size, which might have restricted our ability to detect significant associations. Generalizability of our findings may be limited, given that the study cohort only included low-income Head Start families. Furthermore, the study design does not allow us to infer causality or test whether associations may be bidirectional.

## Conclusion

Including multiple family members in child obesity programs can be associated with more positive outcomes [2]; including siblings as part of family-based programs represents a novel approach. Future studies are needed to further explore the role of siblings in feeding and the effect of including them in obesity prevention interventions.
